# Health Outcome Changes in Individuals With Type 1 Diabetes After a State-Level Insulin Copayment Cap

**DOI:** 10.1001/jamanetworkopen.2024.25280

**Published:** 2024-08-14

**Authors:** Theodoros V. Giannouchos, Benjamin Ukert, Thomas Buchmueller

**Affiliations:** 1 Department of Health Policy and Organization, School of Public Health, The University of Alabama at Birmingham; 2 Center for Outcomes and Effectiveness Research and Education, Heersink School of Medicine, The University of Alabama at Birmingham; 3 Department of Health Policy and Management, School of Public Health, Texas A&M University, College Station; 4 Stephen M. Ross School of Business, University of Michigan, Ann Arbor; 5 Office of the Assistant Secretary for Planning and Evaluation, US Department of Health and Human Services, Washington, DC

## Abstract

**Importance:**

Many insulin users ration doses due to high out-of-pocket costs. Starting January 2020 with Colorado, 25 states and the District of Columbia enacted laws that cap insulin copayments.

**Objective:**

To estimate the association of Colorado’s $100 copayment cap with out-of-pocket spending, medication adherence, and health care services utilization for diabetes-related complications.

**Design, Setting, and Participants:**

In this cohort study using Colorado’s All-Payer Claims Database, nonelderly insulin users with type 1 diabetes were analyzed from January 2019 to December 2020. Outcome changes were compared in the prepolicy and postpolicy period among individuals continuously enrolled in state-regulated and non–state-regulated plans using difference-in-differences regressions. Subgroup analyses were conducted based on individuals’ prepolicy spending (low: never ≥$100 out-of-pocket vs high: ≥$100 out-of-pocket cost at least once). Data were analyzed from June 2023 to May 2024.

**Exposure:**

Enrollment in state-regulated health insurance plans subject to the copayment cap legislation.

**Main Outcomes and Measures:**

Adherence to basal and bolus insulin treatment was evaluated using the proportion of days covered measure, out-of-pocket spending reflected prescription cost for a 30-day supply, and health care utilization for diabetes-related complications was identified using primary diagnosis codes from medical claims data.

**Results:**

The panel included 1629 individuals with type 1 diabetes (39 096 person-months), of which 924 were male (56.7%), 540 (33.1%) had 1 or more comorbidities, and the mean (SD) age was 40.6 (15.9) years. Overall, the copayment cap was associated with out-of-pocket spending declines of $17.3 (95% CI, −$27.3 to −$7.3) for basal and $11.5 (95% CI, −$24.7 to $1.7) for bolus insulins and increases in adherence of 3.2 (95% CI, 0.0 to 6.5) percentage points for basal and 3.3 (95% CI, 0.3 to 6.4) percentage points for bolus insulins. Changes in adherence were associated with increases within the prepolicy high-spending group (basal, 9.9; 95% CI, 2.4 to 17.4 percentage points; bolus, 13.0; 95% CI, 5.1 to 20.9 percentage points). The policy was also associated with a mean reduction of −0.09 (95% CI, −0.16 to −0.02) medical claims for diabetes-related complications per person per month among high spenders, a 30% decrease.

**Conclusions and Relevance:**

In this cohort study of Colorado’s insulin copayment cap among individuals with type 1 diabetes, the policy was associated with an overall decline in out-of-pocket spending, an increase in medication adherence, and a decline in claims for diabetes-related complications only among insulin users who spent more than $100 in the prepolicy period at least once.

## Key Points

**Question:**

What is the association of a state-level $100 insulin copayment cap with out-of-pocket spending, treatment adherence, and health care services use for diabetes-related complications?

**Findings:**

In this cohort study of 1629 individuals with type 1 diabetes, out-of-pocket spending declined, treatment adherence increased, and health care services use for diabetes-related complications declined in the postpolicy period only for individuals enrolled in health plans subject to the cap who spent more than the copayment cap in the prepolicy period compared with those not affected by the cap.

**Meaning:**

These findings suggest that improvements in health outcomes through copayment cap policies are contingent on the level of the cap.

## Teaser

This cohort study evaluates the association of a US state copayment cap with out-of-pocket spending, medication adherence, and health care services utilization for diabetes-related complications among individuals with type 1 diabetes.

## Introduction

Diabetes is the most expensive chronic condition in the US, and individuals with diabetes account for almost 1 out of every 4 dollars spent on health care.^1,2^ Approximately 38 million individuals have diabetes, and more than 7 million use 1 or more formulations of insulin.^1,2,3,4^ Of those, around 2 million have type 1 diabetes, which requires lifelong insulin therapy.^3^

Although the cost of insulin products has substantially increased over the past 2 decades, prices for many insulin products have plateaued in recent years due to manufacturer discounts.^5^ Despite this, list prices for insulin products remain high—and about 10 times higher than in most other countries, thus creating severe affordability problems for patients.^5,6^ Lifetime costs of insulin therapy and treatment of long-term complications associated particularly with type 1 diabetes impose a high economic burden on individuals with this condition, with mean type 1 diabetes-related medical costs of $800 per month per person.^7^ Hence, it is not surprising that 1 in 6 of all insulin users with diabetes reports rationing or skipping insulin doses due to high out-of-pocket costs.^8,9,10^ In addition, more than 15% of insulin-dependent individuals with diabetes allocate almost half of their family income to insulin prescriptions.^8,11^

To address the growing out-of-pocket burden, 25 states and the District of Columbia recently enacted laws that cap insulin out-of-pocket payments for individuals covered by state-regulated commercial health insurance plans.^12^ Similar caps have also been implemented in Medicare through the Inflation Reduction Act of 2022.^13,14^ To date, states that have enacted such laws cap out-of-pocket spending to $100 or less for a 30-day supply, but most do not limit copayments for multiple prescriptions.^12^ Colorado was the first state to implement a copayment cap on insulin, with legislation that capped copayments at $100 for a 30-day supply per prescription effective for plans renewed after January 2020.^12,15^

Spending caps on out-of-pocket costs might improve access and adherence to insulin therapies and decrease adverse health outcomes. Previous studies have documented an inverse relationship between out-of-pocket cost and medication adherence, as well as subsequent health outcomes.^16,17,18,19,20,21,22,23,24,25^ Higher cost-sharing has been associated with decreased treatment adherence and discontinuation, adverse health outcomes and increased utilization of inpatient services across various settings, populations, and diseases.^16,17,18,19,20,21,22,23,24,25^ To date, only 2 recent studies using single insurer data have documented declines in out-of-pocket costs but did not find changes in insulin use associated with the implementation of copayment cap policies.^26,27^ In contrast, a study on Medicare’s Inflation Reduction Act cap on cost sharing found increases in the total number of insulin fills.^18^ Despite the important contributions of previous work, empirical evidence regarding the association of the recently enacted insulin cap policies with relevant health outcomes, beyond out-of-pocket costs and fills, has been lacking. To address this gap in the literature, we estimated the association between Colorado’s Insulin Affordability Program and (1) out-of-pocket spending, (2) adherence to insulin therapy, and (3) use of health care services for diabetes-related complications.

## Methods

### Study Design and Data Source

In this cohort study, we used difference-in-differences regression models to estimate the association of the copayment cap policy with changes in out-of-pocket spending, medication adherence, and health care services utilization for diabetes-related complications among insulin-dependent individuals with diabetes in Colorado. We used medical and pharmaceutical claims data from Colorado’s All Payor Claims Database (CO APCD), which contained claims from 40 commercial health insurers.^28^ The data included information on prescription and medical utilization and costs, enrollment, and demographic characteristics. Although self-insured plans that are covered by the Federal Employee Retirement Income Security Act of 1974 (ERISA) are not impacted by the Colorado legislation and are also not required to submit data to state APCDs, the Center for Improving Value in Health Care, the nonprofit organization that manages CO APCD, estimates that the database contains roughly one-quarter of all ERISA plans.^28^ Using individuals’ encrypted identifiers, we constructed a dataset at the person-month level, similar to previous work.^25^ We followed the Strengthening the Reporting of Observational Studies in Epidemiology (STROBE) reporting guideline to report our study.^29^ This study was approved by the institutional review board of Texas A&M University as nonhuman participants research. Informed consent was not required because data were deidentified.

### Study Population

We included individuals who were younger than 65 years and were continuously enrolled in the same type of health plan (state-regulated or not state-regulated) from January 2018 to December 2020, similar to previous work.^26,30^ Individuals who switched between these 2 types of plans, those with a break in enrollment of 1 month or more throughout the study period, and those who died during the study period were excluded (eTable 1 in Supplement 1). We focused only on individuals with type 1 diabetes because these patients require lifelong and continuous insulin therapy, which enabled us to evaluate insulin-treatment adherence with higher accuracy. We excluded individuals with type 2 diabetes because some may not need or stop taking insulin and switch to noninsulin medications, contrary to those with type 1 diabetes. We identified individuals as having type 1 diabetes if they had at least 1 medical claim with a diagnosis for this condition using the *International Statistical Classification of Diseases and Related Health Problems, Tenth Revision* codes (eTable 2 in Supplement 1 lists the codes). We required at least 1 prescription fill for ultralong, long, or intermediate-acting insulin (basal) and at least 1 prescription fill for rapid-acting or short-acting insulin (bolus) within 1 year before January 2019 (index date) for inclusion in the sample a priori, since most individuals with type 1 diabetes require a combination of basal and bolus insulin (eTable 1 in Supplement 1). This restriction enabled us to identify individuals who were already using both types of insulin before the index month, and thus create a balanced panel with more accurate baseline outcome estimates. Insulin-related pharmacy claims were identified using National Drug Codes (eTable 3 in Supplement 1 lists the codes).

### Outcome Measures

We defined the exposure using an interaction term indicating whether an individual was enrolled in state-regulated health insurance plans which were subject to the cap effective January 1, 2020, in the postpolicy implementation period (January 2020 to December 2020). Our main outcomes of interest were individuals’ out-of-pocket spending, adherence to insulin therapy, and utilization of health care services for diabetes-related complications. Out-pocket-spending was calculated using individuals’ copayment and cost-sharing amounts from the claims data and adjusted to reflect cost for a 30-day supply for each type of insulin prescription. We used the proportion of days covered to measure medication adherence for each type of insulin, which adjusts for overlapping days of supply, and thus has distinct advantages over other commonly used adherence metrics. Treatment adherence was measured separately for basal and bolus insulins. We also identified all medical claims (inpatient and outpatient) for common diabetes-related complications (eg, hyperglycemia, hypoglycemia, ketoacidosis, tissue or skin infections, retinopathy, cardiovascular diseases, and kidney complications) using the primary diagnosis code for each claim and aggregated those at the person-month level (eTable 4 in Supplement 1 lists the codes). In addition, since some complications will manifest over longer time periods than our 2-year study period, we also explored claims for short-term diabetes-related complications only.^31^ Since we anticipated that the cap would have differential outcomes depending on individuals’ level of spending, we further stratified insulin users into 2 subgroups based on whether they had at least 1 insulin-related pharmaceutical claim with out-of-pocket spending of $100 or more in the prepolicy period (high spenders) or not (low spenders). We also validated potential policy-associated changes by exploring changes in noninsulin prescriptions among the included individuals. Individuals were followed over 24 months (January 2019 to December 2020) and the unit of analysis was at the person-month level.

### Covariate Measures

We included individual-level measures of age groups, sex, residence in a rural area, and a baseline comorbidity index, similar to previous work.^25^ We used the Elixhauser comorbidity index to construct a comorbidity score using diagnoses from all medical claims in the 12 months before the index date of January 1, 2019. We also included a dichotomous variable for the type of plan that individuals were enrolled in (high-deductible health plan with a health savings accounts [HDHP] or not) and indicators for the health service areas where individuals live. Finally, we also included a dichotomous indicator for having any additional medical claims in a given month beyond diabetes since these might impose financial strains and affect prescription fills.

### Statistical Analysis

We initially conducted a descriptive analysis of all insulin users with type 1 diabetes stratified by health plan type (state-regulated or not) at the index month (January 2019). We compared all outcomes and individual characteristics using χ^2^ tests for categorical and *t* tests for numerical variables. To estimate the policy’s association with out-of-pocket spending, treatment adherence, and diabetes-related complications, we used a difference-in-differences generalized linear regression model, controlling for all covariates mentioned previously as well as individual fixed-effects given the panel structure of our data. Our independent variable of interest was an interaction term between 2 dichotomous variables indicating whether an individual was enrolled in a state-regulated health insurance plan that was affected by the copayment cap policy (treatment group) or not (control group) and whether the period was before or after the cap was in effect. This enabled us to compare changes in outcomes before and after the policy was implemented between affected and nonaffected individuals in each month. Regression models were conducted separately for out-of-pocket spending and treatment adherence by type of insulin, and then stratified by prepolicy levels of spending among high and low spenders. To account and adjust for seasonal variation in outcomes, particularly since deductibles reset in January and out-of-pocket costs are high until those are met during the year, which can affect prescription filling, we also used indicators for calendar month, in addition to the covariates mentioned previously. We evaluated prepolicy trend differences to examine whether modeling assumptions (parallel trends) hold using an event study by interacting the treatment indicator with each month in 2019. We also conducted sensitivity analyses by excluding 2 months before and 2 months after the policy was implemented to limit anticipatory behaviors and spillover outcomes (eg, not filling prescriptions immediately before the policy) and to evaluate the robustness of our findings. Finally, we also conducted a falsification test by replicating the regression model using noninsulin prescriptions as the outcome to validate our findings. SEs were robust to heteroskedasticity. Significance was set at a *P *value less than .05. Data were managed using SAS version 9.0 (SAS Institute) and statistical analyses were conducted in Stata version 16.0 (StataCorp). Data were analyzed from June 2023 to May 2024.

## Results

The final sample included 1629 insulin users (39 096 person-months) with type 1 diabetes (Table 1). At the baseline month, the mean (SD) age was 40.6 (15.9) years, 924 (56.7%) were male, and 540 had at least 1 comorbidity beyond diabetes (21.9% had 1; 11.2% had 2 or more). A total of 123 (7.6%) were enrolled in HDHPs and 337 insulin users (20.7%) had at least 1 medical claim for a diabetes-related complication. Overall, only about 1 in 4 insulin users had at least 1 prescription for basal or bolus insulin that exceeded $100 in out-of-pocket spending at any month in the prepolicy period. The mean (SD) out-of-pocket spending for a 30-day supply at baseline was $67.8 (95% CI, $60.3-$80.3) for basal insulins and $87.5 (95% CI, $74.9-$100.0) for bolus insulins. Mean treatment adherence was 63.3% (95% CI, 61.0%-65.6%) for basal insulins and 64.7% (95% CI, 62.4%-66.9%) for bolus insulins at baseline. Most differences between the treatment and control group in the baseline month were small and not statistically significant.

**Table 1.** Baseline Characteristics of Insulin Users With Type 1 Diabetes in January 2019

Characteristics	Patients, No. (%)			*P* value
	Total (n = 1629)	Non–state-regulated plans (n = 272)	State-regulated plans (n = 1357)	
No. of person-months	39 096	6528	32 568	NA
Individual characteristics				
Sex				
Female	705 (43.3)	123 (45.2)	582 (42.9)	.50
Male	924 (56.7)	149 (54.8)	775 (57.1)	
Age, y				
Mean (SD)	40.6 (15.9)	42.2 (15.4)	40.2 (15.9)	.06
Groups				
0-18	189 (11.6)	26 (9.6)	163 (12.0)	.26
19-24	145 (8.9)	26 (9.6)	119 (8.8)	
25-44	522 (32.0)	78 (28.7)	444 (32.7)	
45-64	773 (47.5)	142 (52.2)	631 (46.5)	
Residence in rural area	201 (12.3)	74 (27.2)	127 (9.4)	<.001
Comorbidities				
None	1089 (66.9)	173 (63.6)	916 (67.5)	.25
1	357 (21.9)	61 (22.4)	296 (21.8)	
≥2	183 (11.2)	38 (14.0)	145 (10.7)	
Most prevalent comorbidities				
Hypertension	143 (8.8)	33 (12.1)	110 (8.1)	.045
Hypothyroidism	121 (7.4)	29 (10.6)	92 (6.8)	.03
Depression	97 (6.0)	16 (5.9)	81 (6.0)	.96
Kidney failure	72 (4.4)	11 (4.0)	61 (4.5)	.87
COPD	70 (4.3)	12 (4.4)	58 (4.3)	.87
High deductible health plan	123 (7.6)	29 (10.6)	94 (6.9)	.04
Had any medical claim during any month	919 (56.4)	150 (55.2)	769 (56.7)	.69
Noninsulin pharmacy claims				
≥1	1040 (63.8)	180 (66.2)	860 (63.4)	.41
Mean (95% CI)	2.5 (1.9-2.2)	2.5 (2.1-2.8)	2.0 (1.9 − 2.1)	.03
≥1 OOP of >$100 prepolicy				
Basal insulin prescription	375 (23.0)	60 (22.1)	315 (23.2)	.75
Bolus insulin prescription	409 (25.1)	65 (23.9)	345 (25.4)	.65
Outcomes in baseline month				
Adherence per person, mean (95% CI)				
Basal insulin	63.3 (61.0-65.6)	65.6 (60.2-71.0)	62.8 (60.3-65.3)	.37
Bolus insulin	64.7 (62.4-66.9)	63.5 (57.9-69.0)	64.9 (62.5-67.4)	.63
OOP spending for 30-d supply per person, mean (95% CI), $US				
Basal insulin	67.8 (60.3-80.3)	55.8 (39.8-71.9)	70.3 (60.3-80.3)	.22
Bolus insulin	87.5 (74.9-100.0)	62.4 (42.6-82.2)	92.9 (78.2-107.6)	.07
Medical claims for diabetes-related complications				
Any short- or long-term				
≥1	337 (20.7)	37 (13.6)	300 (22.1)	.001
Mean (95% CI)	0.33 (0.30-0.37)	0.23 (0.14-0.31)	0.35 (0.31-0.40)	.02
Short-term only				
≥1	277 (17.0)	35 (12.9)	242 (17.8)	.05
Mean (95% CI)	0.26 (0.23-0.29)	0.20 (0.13-0.28)	0.27 (0.23-0.31)	.11

Abbreviations: COPD, chronic obstructive pulmonary disease; OOP, out-of-pocket.

**Figure 1.**
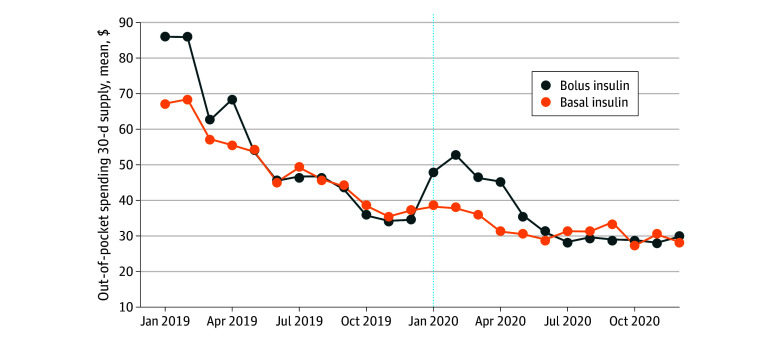
Trends in Mean Monthly Out-of-Pocket Spending per Person for 30-Day Supply Among All Insulin Users With Type 1 Diabetes From January 2019 to December 2020 Vertical line indicates the implementation month of the copayment cap policy (January 2020).

Overall, the findings for mean monthly out-of-pocket spending for a 30-day supply per individual suggested seasonal patterns (Figure 1). Out-of-pocket spending for both basal and bolus insulin was high in January and February and low over the last few months across both years, corresponding to periods when individuals reach their deductibles or out-of-pocket maximums. However, out-of-pocket spending was lower after January 2020, the month of the copayment cap policy implementation, relative to the corresponding prepolicy months, and seasonal variation was attenuated, particularly for basal insulin.

**Figure 2.**
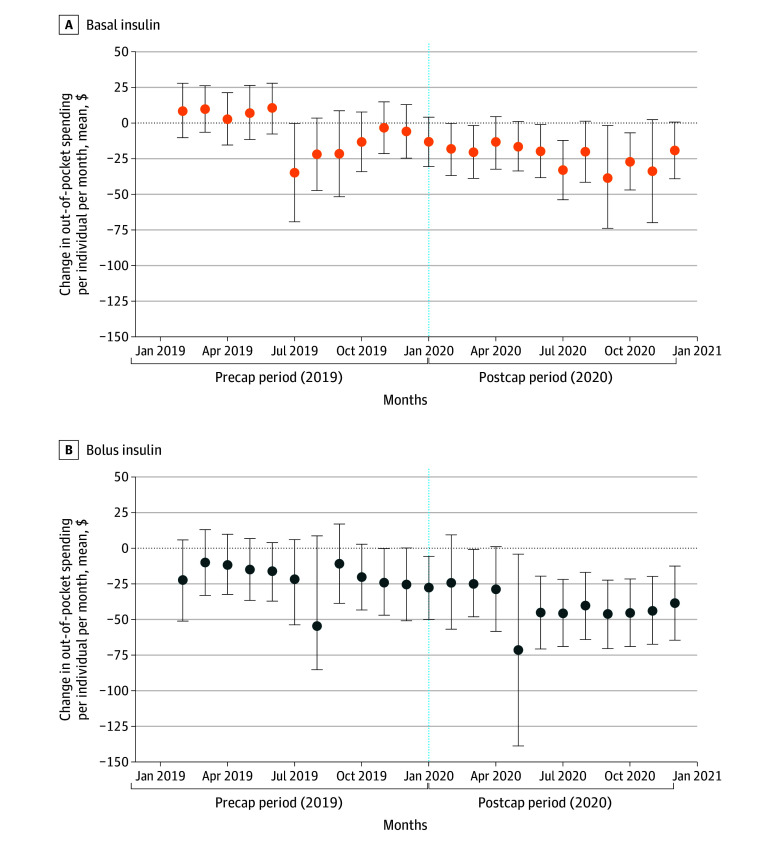
Event Study Estimates of Changes in Out-of-Pocket Spending Among All Insulin Users With Type 1 Diabetes Points represent estimated coefficients indicating differences between the treatment and control group in each month from the event study regressions (with January 2019 as the reference group). Vertical line indicates the implementation month of the copayment cap policy (January 2020). Error bars indicate 95% CIs.

**Figure 3.**
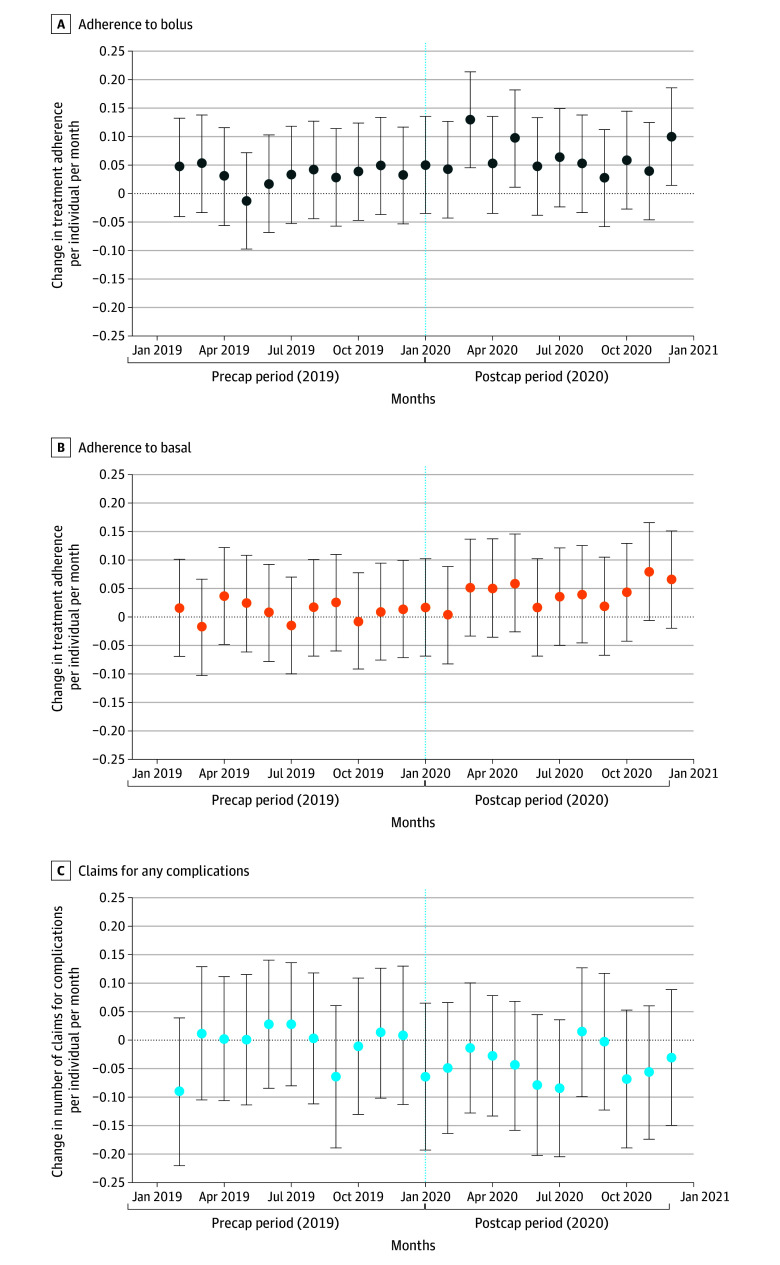
Event Study Estimates of Changes in Treatment Adherence and Medical Claims for Diabetes-Related Complications Among All Insulin Users With Type 1 Diabetes Points represent estimated coefficients indicating differences between the treatment and control group in each month from the event study regressions (with January 2019 as the reference group). Vertical line indicates the implementation month of the copayment cap policy (January 2020). Error bars indicate 95% CIs.

Out-of-pocket spending declined by a mean of $20.7, from $51.8 to $31.1, for basal insulins and by $18.3, from $54.2 to $35.9, for bolus insulins per person per month for those enrolled in state-regulated plans in the postpolicy period (Table 2). The regression-adjusted model showed a significant policy-associated decline in mean monthly out-of-pocket spending for a 30-day supply per prescription by $17.3 (95% CI, −$27.3 to −$7.3; a decrease of 33%) for basal insulins and by $11.5 (95% CI, −$24.7 to $1.7; a decrease of 21%) for bolus insulins (Figure 2). Declines in out-of-pocket spending were larger for individuals who had spent more than $100 out-of-pocket at least once in the prepolicy period. However, within this high-spending subgroup, out-of-pocket spending also declined for those enrolled in non–state regulated health plans in the postpolicy period, but the decline was smaller than for those enrolled in state-regulated health plans. Adherence for basal insulins remained relatively similar in the prepolicy and postpolicy periods among those affected by the policy (treatment group: from 53.4% to 53.0%) but declined by 3.2 percentage points among those who were not subject to the $100 cap (control group: from 59.1% to 55.9%) (Table 2). This resulted in a significant regression-adjusted increase in adherence for basal insulins by 3.2 (95% CI, 0.0 to 6.5) percentage points for individuals in state-regulated health plans, a 6% increase (Table 2, Figure 3). Among bolus insulins, adherence was relatively similar in the prepolicy and postpolicy periods for insulin users in the control group (from 58.2% to 57.9%) but increased by 4.2 percentage points (from 61.4% to 65.6%) in the postpolicy period for those in state-regulated plans. The regression-adjusted model showed a significant policy-associated increase in adherence by 3.3 (95% CI, 0.3 to 6.4) percentage points for bolus insulins, a 5% increase.

**Table 2.** Difference-in-Differences Regression Model Estimates of Changes in Out-of-Pocket Spending, Treatment Adherence, and Medical Claims for Diabetes-Related Complications Among All Insulin Users With Type 1 Diabetes by Insulin Type Overall and by Levels of Prepolicy Spending

Analysis	Mean value		Difference	Difference-in-differences	
	Precap period	Postcap period		Unadjusted	Adjusted (95% CI)
Overall					
Treatment adherence^a^					
Basal insulins					
Control group	59.1	55.9	−3.2	NA	NA
Treatment group	53.4	53.0	−0.4	2.8	3.2 (0.0 to 6.5)
Bolus insulins					
Control group	58.2	57.9	−0.3	NA	NA
Treatment group	61.4	65.6	4.2	4.5	3.3 (0.3 to 6.4)
Claims for complications, No.					
Any short- or long-term					
Control group	0.17	0.17	0.00	NA	NA
Treatment group	0.32	0.29	−0.03	−0.03	−0.04 (−0.07 to 0.00)
Short-term only					
Control group	0.16	0.16	0.00	NA	NA
Treatment group	0.25	0.22	−0.03	−0.03	−0.02 (−0.04 to 0.01)
Out-of-pocket spending, $US					
Basal insulins					
Control group	40.8	36.8	−4.0	NA	NA
Treatment group	51.8	31.1	−20.7	−16.7	−17.3 (−27.3 to −7.3)
Bolus insulins					
Control group	47.3	37.6	−9.7	NA	NA
Treatment group	54.2	35.9	−18.3	−8.6	−11.5 (−24.7 to 1.7)
High spenders					
Treatment adherence^a^					
Basal insulins					
Control group	65.0	58.6	−6.4	NA	NA
Treatment group	53.4	54.7	1.3	7.7	9.9 (2.4 to 17.4)
Bolus insulins					
Control group	61.2	55.3	−5.9	NA	NA
Treatment group	64.1	69.5	5.4	11.3	13.0 (5.1 to 20.9)
Claims for complications, No.					
Any short- or long-term					
Control group	0.16	0.21	0.05	NA	NA
Treatment group	0.28	0.25	−0.03	−0.08	−0.09 (−0.16 to −0.02)
Short-term only					
Control group	0.16	0.19	0.03	NA	NA
Treatment group	0.22	0.19	−0.03	−0.06	−0.05 (−0.11 to 0.00)
Out-of-pocket spending, $US					
Basal insulins					
Control group	88.9	47.7	−41.2	NA	NA
Treatment group	119.4	42.8	−76.6	−35.4	−43.2 (−74.0 to −12.4)
Bolus insulins					
Control group	111.8	60.2	−51.6	NA	NA
Treatment group	117.4	56.3	−61.1	−9.5	−18.4 (−60.9 to 24.1)
Low spenders					
Treatment adherence^a^					
Basal insulins					
Control group	57.7	55.3	−2.4	NA	NA
Treatment group	53.4	52.5	−0.9	1.5	1.3 (−2.4 to 4.9)
Bolus insulins					
Control group	57.6	58.5	1.2	NA	NA
Treatment group	60.4	64.2	3.8	2.6	0.0 (−0.3 to 0.3)
Claims for complications, No.					
Any short- or long-term					
Control group	0.17	0.17	0.00	NA	NA
Treatment group	0.32	0.30	−0.02	−0.02	−0.02 (−0.06 to 0.02)
Short-term only					
Control group	0.17	0.15	−0.02	NA	NA
Treatment group	0.26	0.23	−0.03	−0.01	0.00 (−0.03 to 0.02)
Out-of-pocket spending, $US					
Basal insulins					
Control group	22.1	32.1	10.0	NA	NA
Treatment group	27.8	27.0	−0.8	−10.8	−9.7 (−15.3 to −4.0)
Bolus insulins					
Control group	21.7	29.3	7.6	NA	NA
Treatment group	28.2	27.1	−1.1	−8.7	−8.4 (−12.2 to −4.7)

Abbreviation: NA, not applicable.

^a^ Precap and postcap values for treatment adherence are given as percentages; difference and difference-in-differences values are given as percentage points.

The overall association of the policy with the increase in adherence to both basal and bolus insulins was entirely attributed to insulin users in the treatment group who had paid $100 or more out-of-pocket at least once in the prepolicy period (Table 2; eFigure 1 and eFigure 2 in Supplement 1). The adjusted regression model showed an increase in adherence of 9.9 (95% CI, 2.4 to 17.4) percentage points for basal insulin and an increase in adherence of 13.0 (95% CI, 5.1 to 20.9) percentage points for bolus insulin among high spenders in the treatment group compared with their corresponding control group, representing increases of around 20%. Changes in adherence for both types of insulin were not statistically significant within the prepolicy low-spending subgroup (never $100 or more) (Table 2; eFigure 2 in Supplement 1).

Medical claims per individual per month for any diabetes-related complications remained unchanged between the prepolicy and postpolicy periods among those who were not subject to the $100 cap but decreased by 0.03 claims per individual per month for those affected by the policy (treatment group: 0.32 to 0.29 claims) (Table 2). The regression estimate showed that the copayment cap was associated with significant reductions in the number of medical claims for diabetes-related complications by a mean of −0.04 (95% CI, −0.07 to 0.00) per individual per month (Table 2, Figure 3). Similar to the changes in adherence, the decline in claims for complications was associated with changes among the prepolicy high-spending group (Table 2; eFigure 1 and eFigure 2 in Supplement 1). Among this subgroup, the copayment cap policy was associated with a mean reduction of −0.09 (95% CI, −0.16 to −0.02) claims for diabetes-related complications per individual per month, a relative reduction of about 30% and equal to about 380 complications averted overall in the postpolicy period, while no significant change was observed within the prepolicy low-spending subgroup (−0.02 claims; 95% CI, −0.06 to 0.02 claims). Estimates for changes in short-term diabetes-related complications were similar (Table 2).

Overall, prepolicy outcomes were relatively similar between the 2 groups (Figure 2 and Figure 3; eFigure 1 and eFigure 2 in Supplement 1). The sensitivity analysis excluding 2 months before and 2 months after the policy yielded similar results (eTable 5 in Supplement 1). Estimates of our falsification analysis using noninsulin prescriptions as the outcome yielded statistically insignificant results, thus further validating our findings (eTable 6 in Supplement 1).

## Discussion

In this cohort study of insulin users with type 1 diabetes in Colorado, we found that state legislation capping out-of-pocket costs at $100 per prescription for a 30-day supply of insulin was associated with declines in out-of-pocket spend and increases in adherence to insulin therapy. However, the overall observed increase in treatment adherence was concentrated only among insulin users who spent $100 or more out-of-pocket at least once in the prepolicy period for a 30-day prescription. Similarly, we found declines in the mean number of medical claims per person per month for diabetes-related complications only among those who spent more than the copayment cap in the prepolicy period.

Our results are in line with previous studies that have examined the outcomes of state-specific and insurer-specific copayment limits and found reductions in out-of-pocket spending, increased adherence to treatments, and fewer adverse outcomes that required the use of health care services.^17,18,19,20,21,22,23,24,25,26,27^ For individuals who require chronic use of expensive medications, such as those with type 1 diabetes, policies that protect these patients from high out-of-pocket spending can result in significant improvements in health outcomes by increasing adherence to clinically important medications. While concerns are raised about potential premium increases due to cost-shifting from patients to insurers, these could be offset by improved treatment adherence and thus lower use of acute and costly medical services due to disease-related complications, as documented in our study. Hence, our empirical evidence supports policymakers’ intentions to make insulin more affordable and in turn to improve access and adherence to treatment for insulin-dependent individuals and their health outcomes.

However, the effectiveness of any policy targeting out-of-pocket spending depends on the level of the copayment cap.^26^ In the case of Colorado, our findings suggest that only about 1 in every 4 insulin users spent $100 or more in the prepolicy period at least in 1 month for a prescription. Similarly, the nationwide mean of out-of-pocket prescription costs is between $40 to $70 per 30-day insulin fill, and thus well below the $100 cap of Colorado.^26,32,33^ Not surprisingly, policy-related changes in adherence and complications in our study were concentrated solely within the high-spending group of insulin users, since most insulin users had generous enough health plans that did not expose them to high out-of-pocket insulin costs. This finding is in line with a study by Garabedian and colleagues^26^ who found that state-level insulin out-of-pocket caps were associated with reduced out-of-pocket costs, particularly in states with lower monetary caps and among individuals enrolled in HDHPs. The legislation evaluated in our study was amended and effective in January 2022, capped out-of-pocket spending on insulin to $100 for a 30-day supply for all prescriptions and added access to 1 emergency prescription within a 12-month period at a cost not to exceed $35 for a 30-day supply for eligible individuals.^34^ Thus, these changes could have expanded access to insulin for more individuals with diabetes and warrant future studies.

Interestingly, we also found reductions in out-of-pocket spending in the postpolicy year among insulin users enrolled in health plans not regulated by the state, which were not subject to the cap. This was also documented in a recent study using data from a single national insurer and a 9-month postpolicy follow-up period.^26^ Reductions in out-of-pocket spending among enrollees of non–state regulated health plans could be attributed to using less costly insulins, switching to health plans with lower deductibles or copayments for pharmaceuticals, or employers voluntarily changing health plan generosity, suggesting the need for future studies. Some large insulin manufacturers have also reacted to the enacted copayment caps by lowering their products’ costs by 70% and capping users’ out-of-pocket spending for their products.^35^ These unintended consequences, which might bias our estimates downwards, are particularly important from a societal welfare and equity standpoint since the spillover effects of the copayment cap policies can yield substantial gains in health outcomes across a broader-than-targeted range of patients.

The implications of our findings are particularly important as many states have currently imposed caps ranging from $25 to $100 for a 30-day supply and can be used to inform initiatives for future adjustments. Studies using data from other states with different levels of copayment caps are needed to provide a more comprehensive view of the impact of such policies on adherence and health outcomes beyond costs. Future work should also evaluate longer-term health outcomes among insulin users and changes in health care services utilization, as well as plan-paid prescription and medical claims costs. Despite this, our findings are timely as states without copayment caps are considering such policies for insulins, and nationwide efforts are under way to cap out-of-pocket insulin costs for all patients with diabetes in the US, with the majority of the public supporting federal regulation to limit out-of-pocket spending for insulin.^34,35,36,37,38^

### Limitations

Our study has several limitations. First, our analysis was limited to Colorado and might not be generalizable to other states, particularly those that also implemented different levels of copayment caps. Second, we evaluated changes in the first year following the policy implementation, and thus future work is needed using more postpolicy years on various relevant outcomes. Third, although differences in prepolicy trends between insulin users in state-regulated and in non–state regulated plans were generally not significant, confounding is possible. Fourth, while proportion of days covered is a commonly used measure of adherence in claims data, it might still be inaccurate if individuals are adjusting or using different dosing that was estimated using the prescriptions’ supply length, which was available in the data. We also note that, due to spillovers, our estimates might be conservative and understate the magnitude of the policy-related changes in the outcomes studied. We also studied only a subset of all insulin-dependent individuals with diabetes due to the prespecified inclusion criteria. However, continuous enrollment over the study period and a history of insulin use before 2019 enabled us to reduce biases due to population changes and to estimate policy-related changes in the outcomes more accurately. Additionally, we note that our findings could further underestimate the policy’s impact since we evaluated the policy only for 2020, the first year of the COVID-19 pandemic. The pandemic might have disproportionately affected the income of individuals in state-regulated plans compared with those in more generous self-insured plans who already faced lower out-of-pocket copayments. Future work is needed to explore and account for individual financial situations, which predisposes adherence to treatment.

## Conclusions

In this cohort study of Colorado’s insulin copayment cap among individuals with type 1 diabetes, our results suggest that the policy targeting and imposing caps on out-of-pocket spending for insulin was associated with declines in out-of-pocket spending and improved treatment adherence and health outcomes, but mostly for individuals with prepolicy spending levels above the cap. These findings can inform states that have recently implemented similar policies and those that are currently considering out-of-pocket caps about the short-term outcomes of the copayment cap legislation.

## Notes

Supplement 1. **eTable 1. **Flow Chart for Study Sample **eTable 2. ***International Statistical Classification of Diseases and Related Health Problems, Tenth Revision* Codes for Type 1 Diabetes **eTable 3. **National Drug Code Codes for Insulins **eTable 4. ***ICD-10* Codes for Diabetes-Related Complications **eTable 5. **Sensitivity Analyses Excluding 2 Prepolicy (November and December 2019) and 2 Postpolicy (January and February 2020) Months **eTable 6. **Falsification Test Using Noninsulin Prescriptions as the Outcome **eFigure 1. **Event Study Estimates of Changes in Treatment Adherence and Medical Claims for Diabetes-Related Complications Among Insulin Users With Type 1 Diabetes Who Spent More Than $100 at Least Once (High-Spenders) in the Prepolicy Period **eFigure 2. **Event Study Estimates of Changes in Treatment Adherence and Medical Claims for Diabetes-Related Complications Among Insulin Users With Type 1 Diabetes Who Never Spent More Than $100 at Least Once (Low-Spenders) in the Prepolicy Period

Supplement 2.
Data Sharing Statement
