# Supplementary material for: Health Outcome Changes in Individuals With Type 1 Diabetes After a State-Level Insulin Copayment Cap
Source: JAMA Netw Open. 2024 Aug 14;7(8):e2425280. doi: 10.1001/jamanetworkopen.2024.25280 (PMC11325206; doi:10.1001/jamanetworkopen.2024.25280)
Supplement: Supplement 1. — eTable 1. Flow Chart for Study Sample eTable 2. International Statistical Classification of Diseases and Related Health Problems, Tenth Revision Codes for Type 1 Diabetes eTable 3. National Drug Code Codes for Insulins eTable 4. ICD-10 Codes for Diabetes-Related Complications eTable 5. Sensitivity Analyses Excluding 2 Prepolicy (November and December 2019) and 2 Postpolicy (January and February 2020) Months eTable 6. Falsification Test Using Noninsulin Prescriptions as the Outcome eFigure 1. Event Study Estimates of Changes in Treatment Adherence and Medical Claims for Diabetes-Related Complications Among Insulin Users With Type 1 Diabetes Who Spent More Than $100 at Least Once (High-Spenders) in the Prepolicy Period eFigure 2. Event Study Estimates of Changes in Treatment Adherence and Medical Claims for Diabetes-Related Complications Among Insulin Users With Type 1 Diabetes Who Never Spent More Than $100 at Least Once (Low-Spenders) in the Prepolicy Period [file jamanetwopen-e2425280-s001.pdf]

Supplemental Online Content

Giannouchos TV, Ukert B, Buchmueller T. Health outcome changes in individuals with type 1 diabetes after a state-level insulin copayment cap. *JAMA Netw Open*. 2024;7(8):e2425280. doi:10.1001/jamanetworkopen.2024.25280

eTable 1. Flow Chart for Study Sample

eTable 2. *International Statistical Classification of Diseases and Related Health Problems, Tenth Revision* Codes for Type 1 Diabetes

eTable 3. National Drug Code Codes for Insulins

eTable 4. *ICD-10* Codes for Diabetes-Related Complications

eTable 5. Sensitivity Analyses Excluding 2 Prepolicy (November and December 2019) and 2 Postpolicy (January and February 2020) Months

eTable 6. Falsification Test Using Noninsulin Prescriptions as the Outcome

eFigure 1. Event Study Estimates of Changes in Treatment Adherence and Medical Claims for Diabetes-Related Complications Among Insulin Users With Type 1 Diabetes Who Spent More Than $100 at Least Once (High-Spenders) in the Prepolicy Period

eFigure 2. Event Study Estimates of Changes in Treatment Adherence and Medical Claims for Diabetes-Related Complications Among Insulin Users With Type 1 Diabetes Who Never Spent More Than $100 at Least Once (Low-Spenders) in the Prepolicy Period

This supplemental material has been provided by the authors to give readers additional information about their work.

© 2024 Giannouchos TV et al. *JAMA Network Open.*

eTable 1. Flow Chart for Study Sample

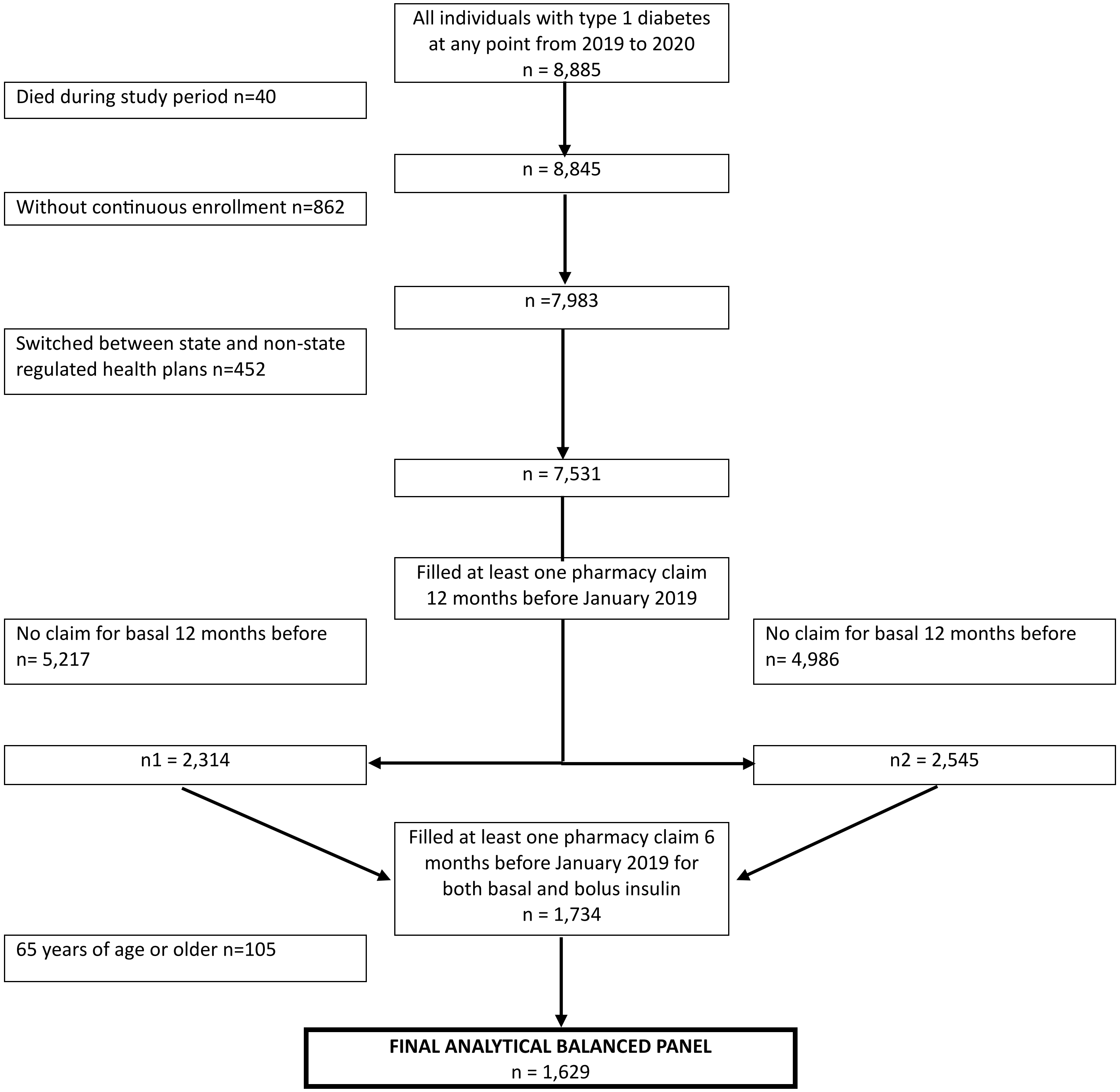

© 2024 Giannouchos TV et al. *JAMA Network Open.*

eTable 2. *International Statistical Classification of Diseases and Related Health Problems, Tenth Revision* (*ICD-10*) Codes for Type 1 Diabetes

E101, E1010, E1011, E102, E1021, E1022, E1029, E103, E1031, E10311, E10319, E103211, E103212, E103213, E103219, E103291, E103292, E103293, E103299, E103311, E103312, E103313, E103319, E103391, E103392, E103393, E103399, E103411, E103412, E103413, E103419, E103491, E103492, E103493, E103499, E103511, E103512, E103513, E103519, E103521, E103522, E103523, E103529, E103531, E103532, E103533, E103539, E103541, E103542, E103551, E103552, E103553, E103559, E103591, E103592, E103593, E103599, E1036, E1037X1, E1037X3, E1037X9, E1039, E104, E1040, E1041, E1042, E1043, E1044, E1049, E1051, E1052, E1059, E106, E10610, E10618, E10620, E10621, E10622, E10628, E10630, E10641, E10649, E1065, E1069, E108, E1085, E109

© 2024 Giannouchos TV et al. *JAMA Network Open.*

eTable 3. National Drug Code Codes for Insulins

00002751001, 00002751017, 00002751099, 00002751101, 00002751199, 00002751201, 00002751299, 00002751601, 00002751659, 00002751699, 00002771201, 00002771227, 00002771261, 00002771299, 00002771401, 00002771459, 00002771461, 00002771501, 00002771559, 00002771563, 00002772601, 00002772605, 00002772801, 00002772899, 00002773701, 00002775201, 00002775205, 00002820701, 00002820705, 00002820761, 00002821301, 00002821305, 00002821401, 00002821405, 00002821501, 00002821517, 00002821599, 00002822201, 00002822259, 00002822801, 00002822827, 00002823301, 00002823301, 00002823305, 00002823305, 00002823501, 00002823505, 00002831501, 00002831517, 00002831599, 00002835101, 00002835105, 00002850101, 00002871501, 00002871517, 00002871599, 00002873001, 00002879701, 00002879759, 00002879761, 00002879799, 00002879801, 00002879859, 00002879899, 00002879901, 00002879959, 00002879961, 00002879999, 00002880301, 00002880359, 00002880361, 00002880501, 00002880559, 00002880599, 00002882401, 00002882427, 00002882459, 00024586900, 00024586901, 00024586903, 00024586905, 00024587100, 00024587101, 00024587102, 2458749000, 00024588236, 00024588463, 00024589463, 00024592410, 00024592500, 00024592501, 00024592505, 00024592605, 00088221900, 00088221901, 00088221905, 00088222033, 00088222034, 00088250033, 00088250034, 00088250200, 00088250201, 00088250205, 00088502000, 00088502001, 00088502005, 00088502101, 00169183302, 00169183311, 00169183402, 00169183411, 00169183702, 00169183711, 00169255013, 00169255090, 00169255097, 00169266015, 00169266090, 00169266097, 00169266211, 00169266290, 00169300301, 00169300312, 00169300315, 00169300325, 00169300401, 00169300412, 00169300415, 00169300425, 00169300701, 00169300712, 00169300715, 00169300725, 00169320111, 00169320190, 00169320415, 00169320490, 00169320497, 00169320511, 00169320515, 00169320515, 00169320591, 00169320595, 00169320595, 00169330312, 00169330390, 00169330391, 00169368512, 00169368592, 00169368712, 00169369619, 00169369690, 00169369697, 00169369698, 00169633810, 00169633890, 00169633898, 00169633910, 00169633990, 00169633997, 00169633998, 00169643810, 00169643890, 00169643897, 00169643898, 00169643910, 00169750111, 00169750190, 00247044710, 00247153810, 00338012612, 35356010200, 47918087490, 47918087890, 47918088018, 47918088236, 47918088463, 47918089190, 47918089463, 47918089818, 47918090218, 49502019580, 49502019671, 49502019673, 49502019675, 49999099310, 49999099410, 50090035200, 50090035209, 50090035300, 50090040300, 50090044400, 50090049700, 50090049800, 50090087600, 50090127600, 50090137500, 50090139800, 50090147500, 50090166300, 50090166400, 50090166500, 50090167800, 50090219300, 50090227200, 50090227300, 50090349100, 50090417700, 50090448800, 50090450100, 5009049550,, 50090495900, 54868142901, 54868274600, 54868347400, 54868361900, 54868438100, 54868462600, 54868510800, 54868520100, 54868532700, 54868532701, 54868576500, 54868583600, 54868588300, 54868589900, 54868605400, 54868623100, 55045350601, 55045360201, 55045368501, 64725075001, 64725183301, 64725183401, 64725183701, 64725222001, 66143751005, 66733077301, 66733082201, 66733082259, 68071170101, 68258889903, 68258897701, 70518138700, 70518138800, 70518138900, 70518139000, 70518146200, 70518186500, 70518211900, 70518225200, 70518226400, 70518239800, 70518239900, 70518280200, 70518280201, 70518282300, 73070010011, 73070010210, 73070010215, 73070010310, 73070010315, 73070020011, 73070020310, 73070020315, 106892, 213442, 242120, 249220, 259111, 260265, 285018, 311026, 311027, 311028, 311033, 311034, 311036, 311040, 311041, 311048, 351297, 351859, 351926, 484322, 485210, 616238, 731281, 752388, 803194, 847187, 847189, 847191, 847199, 847203, 847211, 847213, 847230, 847232, 847239, 847241, 847252, 847254, 847259, 847261, 865098, 977840, 977842, 1543202, 1543207, 1544488, 1544490, 1544568, 1544569, 1544570, 1544571, 1604539, 1604544, 165223, 1652242, 1652639, 1652640, 1652644,

© 2024 Giannouchos TV et al. *JAMA Network Open.*

1652646, 1653196, 1653198, 1653202, 1653204, 1654862, 1654910, 1654912, 1656705, 1656706, 1670011, 1670016, 1670021, 1670023, 1731315, 1731317, 1736863, 1798387, 1798388, 1862101, 1862102, 1926331, 1926332, 1986354, 1986356, 1992169, 1992171, 2002419, 2002420, 2049380, 2100028, 2100029, 2107520, 2107522, 2179744, 2179749, 2205454, 2206090, 2206092, 2206099, 2268064, 2268065, 2377134, 2377231, 2380231, 2380236, 2380248, 2380250, 2380254, 2380256, 2380259, 2380260, 2380265, 2380266, 2380267, 2380268

© 2024 Giannouchos TV et al. *JAMA Network Open.*

eTable 4. *ICD-10* Codes for Diabetes-Related Complications

E1011, E1010,E1310,E1301,E141, E1311, E0810, E0811, E0865, E0800, E1301, E1365, E1300, E0865, E0801, E1065, E102, E1021, E1022, E1029, E09620, E09622, E09628, E1051, E1052, E1351, E1451, E10610, E13610, E1461, E1070, E1371, E1471, E08620, E08621, E08622, E08628, E1031, E10311, E10319, E1032, E103211, E103212, E103213, E103219, E10329, E103291, E103292, E103293, E103299,E10331, E103311, E103312, E103313, E103319, E10339, E10391,E103391, E103392, E103393, E103399, E10392, E10393, E10399, E1034, E103411, E103412, E103413, E103419, E103491, E103492, E103493, E103499, E1035, E10351, E103511, E103512, E103513, E103519, E103521, E103522, E103523, E103529, E103531, E103532 ,E103533, E103539, E103541, E103542, R02, E103551, E103552, E103553, E103559, E10359, E103591, E103592,E103593,E103599, E1036, , E10641, E08311, E08319, E0836, E0839, E1037, E1039, E13319, E133211, E133213, E133291, E133292, E133293, E133299, E133312, E133313, E133319, E133391, E133411, E133412, E133491, E133493, E133499, E133511, E133512, E133513, E133519, E133532, E133551, E133553, E133559, E133591, E133592, E1335, E133593, E133599, E1336, L030, L03011, L03012, L03019, L03021, L03022, L03029, L03031, L03032, L03039, L03041, L03042, L03049, L031, L03111, L03112, L03113, L03114, L03115, L03116, L03119, L03121, L03122, L03123, L03124, L03125, L03126, L03129, L03211, L03212, L03213, L03221, L03222, L03311, L03312, L03313, L03314, L03315, L03316, L03317, L03319, L03321, L03323, L03324, L03327, L03329, L03811, L03818, L03891, L0391, L040, L041, L042, L043, L048, L049, L0501, L0502, L0591, L0592, L080, L081, L0881, L0882, L0889, L089, M726, A480, I21, I210, I2101, I2102, I2109 ,I2111, I2119, I2121 ,I2129, I213, I214, I219, I21A1, I21A9, I220, I221, I222, I228, I229, I200, I201, I208, I209, I501, I502, I5020, I5021, I5022, I5023, I5033, I5040, I5041, I5042, I5043, I508, I50810, I50811, I50812, I50813, I50814, I5082, I5083, I5084, I5089, I509, I610, I611, I612, I613, I614, I615, I616, I618, I619, I6300, I63011, I63012, I63013, I63019, I6302, I63031, I63032, I63033, I63039, I6309, I6310, I63111, I63112, I63113, I63119, I6312, I63131, I63132, I63133, I63139, I6319, I6320, I63211, I63212, I63213, I63219, I6322, I63231, I63232, I63233, I63239, I633, I63311, I63312, I63313, I63319, I63321, I63322, I63323, I63329, I63331, I63332, I63333, I63339, I63341, I63342, I63343, I63349, I6340, I6339, I63411, I63412, I63413, I63419, I63421, I63422, I63423, I63429, I63431, I63432, I63433, I63439, I63441, I63442, I63443, I63449, I6350, I6349, I63511, I63512, I63513, I63519, I63521, I63522, I63523, I63529, I63531, I63532, I63533, I63539, I63541, I63542, I63543, I63549, I636, I638, I6389, I639, G453, G458, G459, R739, R7309, R7302, L00, L0100, L0101, L0103, L0109,L011, L020, L0201, L0202, L0203, L0211, L0212, L0213, L022, L02211, L02212, L02213, L02214, L02215, L02216, L02219, L02221, L02222 ,L02223, L02224, L02225, L02226,L02229, L02231, L02232, L02233, L02234, L02235, L02239, L023, L0231, L0232, L0233, L024, L02411, L02412, L02413, L02414, L02415, L02416, L02419, L02421, L02422, L02423, L02424, L02425, L02426, L02429, L02431, L02432, L02433, L02434, L02435, L02436, L02439, L02511, L02512, L02519, L02521, L02522, L02611, L02612, L02619, L02621, L02622, L02631, L02632, L02811, L02818, L02821, L02828, L02831, L02838, L0291, L0292, L0293, L02431, L02432, E1062, E10620, E10621, E10622, E10628

© 2024 Giannouchos TV et al. *JAMA Network Open.*

eTable 5. Sensitivity Analyses Excluding 2 Prepolicy (11-12/2019) and 2 Postpolicy (01-02/2020) Months

**Analysis**	**Difference-in-Differences**
	Adjusted (95% CI)
**Proportion of Days Covered (%)**	
**Basal insulins**	
Overall	3.8 (0.1 to 7.5)
High spenders	12.3 (4.0 to 20.6)
Low spenders	1.4 (-2.7 to 5.4)
**Bolus insulins**	
Overall	3.9 (0.5 to 7.3)
High spenders	15.1 (6.1 to 24.1)
Low spenders	0.0 (-0.3 to 0.4)
**Claims for complications (avg)**	
**Any short- or long-term**	
Overall	-0.03 (-0.07 to 0.01)
High spenders	-0.09 (-0.16 to -0.02)
Low spenders	-0.01 (-0.06 to 0.03)
**Short-term only**	
Overall	-0.01 (-0.04 to 0.02)
High spenders	-0.04 (-0.08 to 0.00)
Low spenders	0.01 (-0.01 to 0.03)
**Out-of-Pocket Spending**	
**Basal insulins**	
Overall	-18.9 (-30.6 to -7.2)
High spenders	-42.4 (-76.6 to -8.2)
Low spenders	-12.4 (-19.3 to -5.6)
**Bolus insulins**	
Overall	-13.5 (-28.7 to 1.7)
High spenders	-21.9 (-70.3 to 26.4)
Low spenders	-10.1 (-14.4 to -5.8)

© 2024 Giannouchos TV et al. *JAMA Network Open.*

eTable 6. Falsification Test Using Noninsulin Prescriptions as the Outcome

**Analysis**	**Difference-in-Differences**
	Adjusted (95% CI)
Overall	0.10 (-0.04 to 0.25)
High spenders	0.19 (-0.14 to 0.51)
Low spenders	0.08 (-0.08 to 0.23)

© 2024 Giannouchos TV et al. *JAMA Network Open.*

eFigure 1. Event Study Estimates of Changes in Treatment Adherence and Medical Claims for Diabetes-Related Complications Among Insulin Users With Type 1 Diabetes Who Spent More Than $100 at Least Once (High-Spenders) in the Prepolicy Period

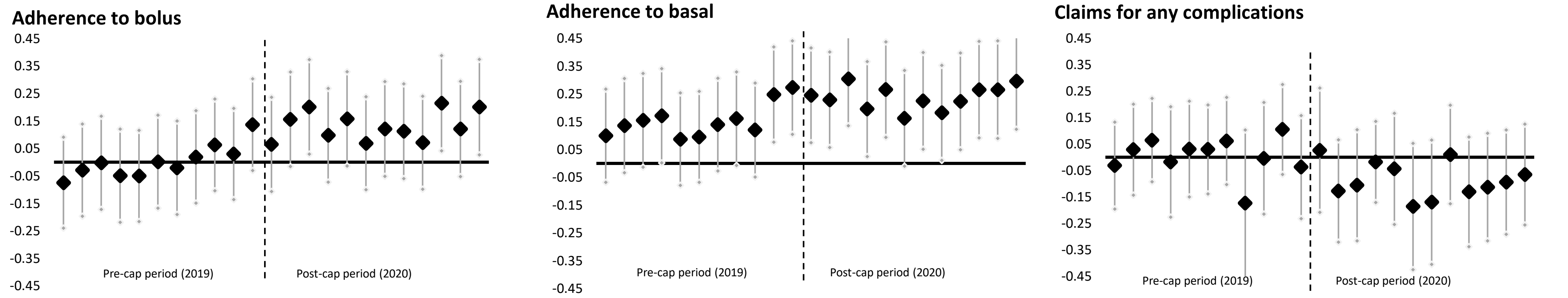

Notes: Symbols present estimated coefficients indicating differences between the treatment and control group in each month from the event study regressions (with January 2019 as the reference group). Vertical line indicates the implementation month of the copay cap policy (01/2020). High spenders are those with at least one insulin prescription fill with out-of-pocket spending of $100 or more in 2019. X-axis: months; Y-axis: adherence – average changes in percentage points on a 0-1 scale per individual per month (e.g. -0.15 indicates a difference of -15pp); Y-axis: claims – changes in the average per individual per month in the number of claims for complications (e.g. -0.15 indicates 0.15 fewer claims per individual per month on average)

© 2024 Giannouchos TV et al. *JAMA Network Open.*

eFigure 2. Event Study Estimates of Changes in Treatment Adherence and Medical Claims for Diabetes-Related Complications Among Insulin Users With Type 1 Diabetes Who Never Spent More Than $100 at Least Once (Low-Spenders) in the Prepolicy Period

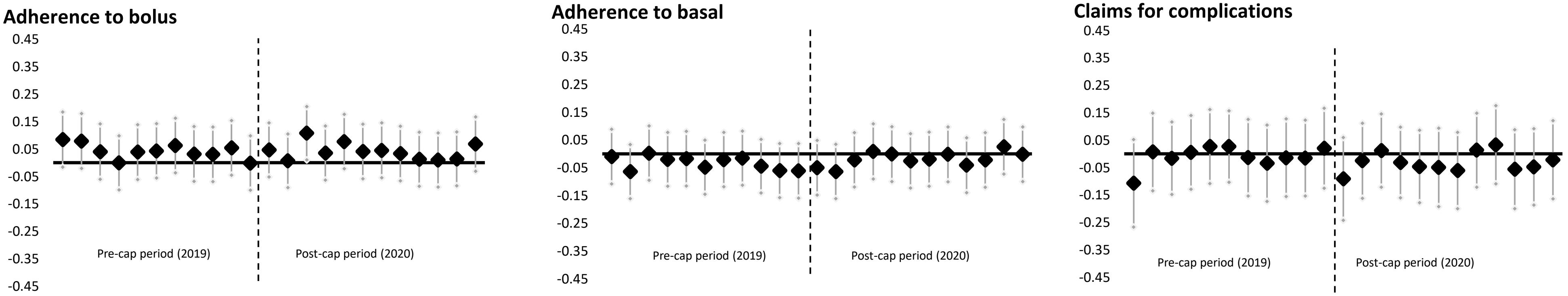

Notes: Symbols present estimated coefficients indicating differences between the treatment and control group in each month from the event study regressions (with January 2019 as the reference group). Vertical line indicates the implementation month of the copay cap policy (01/2020). Low spenders are those who neve paid more than $100 out-of-pocket in 2019. X-axis: months; Y-axis: adherence – average changes in percentage points on a 0-1 scale per individual per month (e.g. -0.15 indicates a difference of -15pp); Y-axis: claims – changes in the average per individual per month in the number of claims for complications (e.g. -0.15 indicates 0.15 fewer claims per individual per month on average)

© 2024 Giannouchos TV et al. *JAMA Network Open.*
