# Supplementary material for: Health Outcome Changes in Individuals With Type 1 Diabetes After a State-Level Insulin Copayment Cap
Source: JAMA Netw Open. 2024 Aug 14;7(8):e2425280. doi: 10.1001/jamanetworkopen.2024.25280 (PMC11325206; doi:10.1001/jamanetworkopen.2024.25280)
Supplement: Supplement 2. — Data Sharing Statement [file jamanetwopen-e2425280-s002.pdf]

Data Sharing Statement

Giannouchos. Health Outcome Changes in Individuals With Type 1 Diabetes After a State-Level Insulin Copayment Cap. *JAMA Netw Open*. Published August 14, 2024. doi:10.1001/jamanetworkopen.2024.25280

Data
Data available: No
